# The clinical effectiveness of the Mind/Body Program for Infertility on wellbeing and assisted reproduction outcomes: a randomized controlled trial in search for active ingredients

**DOI:** 10.1093/humrep/deae119

**Published:** 2024-06-08

**Authors:** Judit Szigeti F, Csaba Kazinczi, Georgina Szabó, Miklós Sipos, Péter Przemyslaw Ujma, György Purebl

**Affiliations:** Institute of Behavioural Sciences, Semmelweis University, Budapest, Hungary; Department of Otorhinolaryngology, Head and Neck Surgery, Semmelweis University, Budapest, Hungary; Department of Clinical Psychology, Semmelweis University, Budapest, Hungary; Doctoral School of Clinical Medicine, University of Szeged, Szeged, Hungary; Doctoral School of Mental Health Sciences, Semmelweis University, Budapest, Hungary; Department of Psychiatry, North Buda Saint John’s Hospital Centre and Outpatient Clinic, Budapest, Hungary; Department of Obstetrics and Gynecology, Assisted Reproduction Centre, Semmelweis University, Budapest, Hungary; Institute of Behavioural Sciences, Semmelweis University, Budapest, Hungary; Institute of Behavioural Sciences, Semmelweis University, Budapest, Hungary

**Keywords:** MAR, infertility-specific distress, mind body programme, pregnancy rate, quality of life, randomized controlled trial, support group, wellbeing

## Abstract

**STUDY QUESTION:**

Does the Mind/Body Program for Infertility (MBPI) perform better, due to certain distinctive elements, than a partly matched support group in improving the wellbeing and medically assisted reproduction (MAR) outcomes of women with elevated distress levels in a clinical setting?

**SUMMARY ANSWER:**

While robust enhancements occurred in the wellbeing overall, the cognitive behavioural and formalized stress management elements of the MBPI allowed a significantly stronger improvement in trait anxiety, but not in other mental health and MAR outcomes, compared with a support group.

**WHAT IS KNOWN ALREADY:**

Mind-body psychological programmes adjacent to MAR have been found to improve women’s mental states and possibly increase chances of pregnancy. However, not enough is known about the programme’s effectiveness among patients with elevated distress levels in routine clinical settings, nor is it clear which of its particular ingredients are specifically effective.

**STUDY DESIGN, SIZE, DURATION:**

A pre-post design, single-centre, randomized controlled trial was performed between December 2019 and October 2022 (start and end of recruitment, respectively). The sample size (n* *=* *168) was calculated to detect superiority of the MBPI in improving fertility-related quality of life. Randomization was computer-based, with random numbers concealing identities of patients until after allocation.

**PARTICIPANTS/MATERIALS, SETTING, METHODS:**

The trial was conducted at a large university teaching hospital. A total of 168 patients were randomly assigned to the mind-body (MBPI) group (n* *=* *84) and the fertility support (FS) control group (n* *=* *84). Patients received a 10-week, 135-min/week group intervention, with the FS group following the same format as the MBPI group, but with a less restricted and systematic content, and without the presumed effective factors. The number of patients analysed was n* *=* *74 (MBPI) and n* *=* *68 (FS) for post-intervention psychological outcomes, and n* *=* *54 (MBPI) and n* *=* *56 (FS) for pregnancy outcomes at a 30-month follow-up.

**MAIN RESULTS AND THE ROLE OF CHANCE:**

Significant improvements occurred in both groups in all psychological domains (adjusted *P *<* *0.001), except for treatment-related quality of life. Linear mixed-model regression analysis did not reveal significantly greater pre-post improvements in the MBPI group than in the FS group in fertility-related quality of life (difference in differences (DD) = 4.11 [0.42, 7.80], *d *=* *0.32, adjusted *P *=* *0.124), treatment-related quality of life (DD = −3.08 [−7.72, 1.55], *d *=* *−0.20, adjusted *P *=* *0.582), infertility-specific stress (DD = −2.54 [−4.68, 0.41], *d *=* *−0.36, adjusted *P *=* *0.105), depression (DD = −1.16 [3.61, 1.29], *d *=* *−0.13, adjusted *P *=* *0.708), and general stress (DD = −0.62 [−1.91, 0.68], *d *=* *−0.13, adjusted *P *=* *0.708), but it did show a significantly larger improvement in trait anxiety (DD = −3.60 [−6.16, −1.04], *d *=* *−0.32, adjusted *P *=* *0.042). Logistic regression showed no group effect on MAR pregnancies, spontaneous pregnancies, or live births.

**LIMITATIONS, REASONS FOR CAUTION:**

The follow-up only covered MAR-related medical outcomes and no psychological variables, and their rates were not equal in the two groups. Biological factors other than age, aetiology, and duration of infertility may have confounded the study results. Loss to follow-up was between 5% and 10%, which may have led to some bias.

**WIDER IMPLICATIONS OF THE FINDINGS:**

The psychologically and medically heterogeneous sample, the normal clinical setting and the low attrition rate all raise the external validity and generalizability of our study. The MBPI works not only in controlled conditions, but also in routine MAR practice, where it can be introduced as a cost-effective, low-intensity psychological intervention, within the framework of stepped care. More studies are needed to further identify its active ingredients.

**STUDY FUNDING/COMPETING INTEREST(S):**

The authors received no financial support for the research, authorship, and/or publication of this article. The authors have no conflict of interest to disclose.

**TRIAL REGISTRATION NUMBER:**

ClinicalTrials.gov NCT04151485.

**TRIAL REGISTRATION DATE:**

5 November 2019.

**DATE OF FIRST PATIENT’S ENROLMENT:**

15 December 2019.

## Introduction

Infertility has significant negative impacts on the lives and mental health of couples facing infertility (World Health Organization, 2023). Research of the past 50 years has not been able to definitively prove that stress can cause infertility (Rooney and Domar, 2018), but the reality of the psychological consequences of unwanted childlessness is widely accepted. According to a resource document of the American Psychiatric Association, up to 40% of women dealing with infertility meet the criteria for a psychiatric diagnosis (Becker *et al.*, 2019), with depressive (Kiani *et al.*, 2021; Nik Hazlina *et al.*, 2022) and anxiety disorders (Kiani *et al.*, 2020) being the most prevalent within clinical samples. On a population level, that is, among people with a history of infertility who do not necessarily seek medical help, levels of psychopathology are lower, but existent (Klemetti *et al.*, 2010; Biringer *et al.*, 2015). Women are generally found to show higher levels of mental health problems connected to infertility than men (Almutawa *et al.*, 2023).

Results have been inconclusive about whether stress in general negatively impacts infertility treatment outcomes (Boivin *et al.*, 2011a; Matthiesen *et al.*, 2011; Nicoloro-SantaBarbara *et al.*, 2018; Peaston *et al.*, 2022), but if the distress reaches mental disorder levels, it seems to slightly interfere with the success of assisted reproductive technology (ART) (Purewal *et al.*, 2018; Zanettoullis *et al.*, 2024). Psychosocial interventions, however, do seem to increase the likeliness of pregnancy and psychological wellbeing in infertility patients (Hämmerli *et al.*, 2009; Frederiksen *et al.*, 2015; Dube *et al.*, 2023), although some meta-analyses have found that the studies lack enough rigour for definitive conclusions about the odds of pregnancy (Kremer *et al.*, 2023).

When invited, only around 5–21% of the clinical population struggling with infertility actually uses psychological help (Boivin *et al.*, 1999; Gray *et al.*, 2014). In a recent international survey, a large disparity was found between the number of respondents reporting an impact of infertility on their mental health and the number who sought psychological support (Boivin *et al.*, 2022). Even so, a vulnerable subgroup of highly distressed patients feel overwhelmed by their infertility, and need psychosocial counselling (Boivin, 1997). However, if unguided, the type of support sought is driven by preference more than its efficacy. The mental health status of patients deteriorates with increasing treatment duration and numbers of unsuccessful attempts (Boivin *et al.*, 2022). Therefore, it is necessary that mental health status be checked at various time points during treatment, and that patients be directed towards evidence-based possibilities for improvement, if need be.

Cognitive behavioural (CB) and mind-body approaches are among the most efficient psychotherapeutic methods in infertility (Katyal *et al.*, 2021; Zhou *et al.*, 2021; Ha *et al.*, 2023). Mind-body methods include, among others, the Mind/Body Program for Infertility (MBPI) (Domar *et al.*, 1990), the Integrative Body-Mind-Spirit programme (Chan *et al.*, 2006, 2012), and mindfulness-based interventions (Galhardo *et al.*, 2013; Nery *et al.*, 2019; Mousavi *et al.*, 2020). The MBPI is a complex intervention, with: (1) ‘mind’ components, such as psychoeducation about the effects of stress and lifestyle on fertility, cognitive therapeutic techniques, assertive communication exercises and self-care concepts, and (2) ‘body’ elements, such as relaxation and meditation techniques and hatha yoga. The programme was introduced as a behavioural treatment (Domar *et al.*, 1990), later called a CB intervention (Domar *et al.*, 2000b), until it gained its final self-definition as a mind-body approach. Meta-analyses, too, have not been uniform in categorizing it, although the actual content has probably not changed much over time.

Two randomized controlled trials (RCTs) on independent samples demonstrated the *efficacy* of the MBPI in terms of both mental health and pregnancy. The first RCT included women trying to conceive for 1–2 years, with any infertility type, cause, or prognosis, but excluded women suffering from clinical depression to avoid the risk of their randomization to the passive control group (Domar *et al.*, 2000a,b). At 1-year follow-up, the study found a significantly higher pregnancy rate in the two intervention (CB and support) groups in relation to the control group, but no difference between the two intervention groups. As for psychological status, while that of the control group deteriorated, both intervention groups ameliorated, and the CB group had significantly better scores than the support group on depression, stress management style and skills, vigour, and self-esteem scales. The second RCT was stricter on biological criteria, with eligible women up to 40 years of age, before their first *in vitro* fertilization (IVF) cycle, and with Day 3 FSH/E2 levels of <12 mIU/ml and <80 pg/ml (respectively), but did not assess mental status, as its only outcome was clinical pregnancy (Domar *et al.*, 2011). Here, the MBPI group showed a significantly higher pregnancy rate in the second IVF cycle than the routine care control group.

Apart from a later feasibility study (Psaros *et al.*, 2015) and an RCT on its internet-based version (Clifton *et al.*, 2020), no further RCTs of the MBPI were done in the USA, nor in other countries. Furthermore, no formal testing has been performed on its *effectiveness*, that is, its ability to induce relevant changes not only in ideal or controlled, but also in normal clinical conditions (Burches and Burches, 2020). It is not known yet how the MBPI can be integrated in the stepped mental health care model of the National Institute for Health and Care Excellence (National Collaborating Centre for Mental Health (UK), 2011) or the psychosocial routine recommended by the European Society of Human Reproduction and Embryology, which suggest that patients in greater distress and/or at risk of emotional problems should be prioritized in receiving specialized care (Boivin, 2002; Gameiro *et al.*, 2015). Finally, since the MBPI is a mixture of different methods not uncommon in the infertility field (Kremer *et al.*, 2023), it is not clear which of its particular ingredients are specifically effective.

The objective of this study was 2-fold: (1) to test the clinical effectiveness of the MBPI on a sample of medically assisted reproduction (MAR)-pursuing women with higher-than-average distress levels, and (2) to shed light on the active ingredients of the programme potentially responsible for its effectiveness. We hypothesized that, due to certain distinctive features, the MBPI outperforms a partly matched fertility support group lacking these elements, for ameliorating distress and possibly contributing toward pregnancies. Thus, the study is partly a replication of Domar *et al.*’ (2000a,b) RCT, but novel in its clinical scope, design, and setting.

## Materials and methods

### Trial design

An interventional, single-centred, two-armed, parallel assignment, RCT with an equal allocation ratio to an experimental group and an active control group was preregistered at Clinical Trials.gov (NCT04151485), consistent with the Consolidated Standards of Reporting Trials (CONSORT) guidelines (Schulz *et al.*, 2010) and the CONSORT extension on reporting social and psychological intervention trials (Grant *et al.*, 2018). The choice of an active control group was justified by its proven superiority over treatment as usual (TAU; Domar *et al.*, 2000a,b). A passive control group was not used for ethical reasons. The study was approved by the Semmelweis University Regional and Institutional Committee of Science and Research Ethics (reference number: 83/2019), Budapest, Hungary, and was carried out in accordance with the tenets of the Declaration of Helsinki.

### Participants and procedures

Women having their first appointments at the Assisted Reproduction Centre of the Department of Obstetrics and Gynaecology of Semmelweis University, Budapest, between December 2019 and October 2022, were consecutively invited via email to participate in mental health screening by filling in a test battery not long after the time of intake (T1) and, if eligible, in the free-of-charge psychological intervention programme. Participation was voluntary, and based on informed consent after learning about the purpose and data management of the research. Questionnaires were completed online, and designed in a way which ruled out missing data. Inclusion criteria were: (1) female sex; (2) reproductive age (18–45 years); (3) meeting the medical criteria for infertility, that is, failure to achieve pregnancy after 12 or more months of regular unprotected sexual intercourse (Zegers-Hochschild *et al.*, 2017); (4) scoring in mild-to-moderate impairment ranges on four psychological tests; and (5) fluency in Hungarian. Women could be in any stage of infertility care from workup to intrauterine insemination (IUI) to IVF with or without intracytoplasmic sperm injection (ICSI). Patients potentially living with substance abuse (Babor *et al.*, 2001; Smith *et al.*, 2010), an eating disorder (Morgan *et al.*, 2000), or experiencing an active psychotic episode (Foussias and Daskalakis, 2019) were excluded from the study, and referred to specialized care. Women scoring in severe ranges on the eligibility tests were referred to individual psychotherapy. Respondents not reaching risk levels on the psychological tests were informed that attending the programme is not essential for them, but they could take the screening tests again at any time in case of perceived deterioration. Those interested in enrolling in the psychological programme despite not presenting a mental issue were referred to groups run by a private care provider, not included in the study sample.

Sociodemographic information such as age, marital status, number of existing children, residence, education, employment status, personal perception of financial situation, health information such as height and weight, and duration, aetiology and treatment stage of infertility were gathered. The trial protocol contained a number of further outcomes, i.e. results of other psychological tests and the intention for treatment continuation, the discussion of which, however, is beyond the scope of this article.

### Sample size

The sample size was estimated using power calculations with G*Power 3 (Faul *et al.*, 2007), requiring an estimated total sample size of 134 to detect a potential significant between-group effect on the primary outcome, that is, fertility quality of life (QoL) as measured by the FertiQoL tool, with 80% power, a 0.1 effect size and a 2-sided *P* < 0.05. The rationale behind aiming for a small effect size was our goal and corresponding design to detect subtle differences between the groups. Based on the literature, an attrition rate of 20% was anticipated (Swift and Greenberg, 2012), raising the total desired recruitment number to 168.

### Randomization

Each eligible patient wishing to join the psychological programme was coded with a 6-digit identification number generated by a statistician using a computer-based random number generator, so that allocation and all further data management and analysis could be anonymized. Patients were then assigned by the psychologist leading the groups with the help of a computer-based random team generator into either a Mind/Body (MBPI) or a Fertility Support (FS) group, with allocation concealment ensured by the use of ID numbers only along the process. Participants, statisticians and medical staff were blinded for intervention allocation. The two types of interventions were performed by the same person; therefore, blinding on the care provider’s part was not possible.

### Interventions

A 20- to 30-min intake interview was conducted by the psychologist leading the groups with every person interested in enrolment, to inform them about the goals, topics and structure of the intervention, and about the randomization process. Additionally, patients were informed about their T1 test results, and were further investigated if the tests signalled states warranting exclusion, i.e. substance abuse, eating disorders, or an active psychotic episode. After randomization, patients were informed about the starting time of their groups and told that, if they cannot adjust to the timetable, they can wait until the next group starts, and go through the randomization process again. Available patients then joined their respective psychological programme, alongside fertility workup and treatment. No pilot study was conducted, since the small sample size allowed by it may not have provided sufficient statistical power to identify with confidence the subtle outcome differences expected. The two types of groups were held in a parallel manner, starting every 3–4 months, with continuous enrolment until the targeted number of participants was reached.

Right before the planned commencement of the programme in February 2020, the first wave of the COVID-19 pandemic broke out. Since the extremity of the situation made programme fidelity impossible, the study was postponed. The patients already enrolled were offered a 10-week online crisis intervention and support programme during the previously unfathomable lockdown, involving the unexpected cessation of ART treatments, with only lifesaving healthcare interventions allowed. The actual onset of the study was in June 2020, when the ART programmes restarted, and the groups were able to meet in person for seven out of ten occasions. The three sessions attended by men, too, continued to be held online for social distancing purposes, because of the potentially double headcount. This ‘7-in-person-3-online’ format was an inevitable diversion from the pre-registered RCT protocol, which was then used for the remainder of the study so that the participants were exposed to unified conditions.

To keep concealment intact, both groups followed the same format of a 10-week, 135-min per session programme. The programme was open for women, with partners also invited for three occasions. Partner non-attendance for any reason was not an exclusion criterion. Groups were held on the same weekday, with group types alternating between afternoons and evenings, i.e. an FS afternoon group and an MBPI evening group in the first round, vice versa in the second, and so on, to avoid bias from schedule clash (typically, afternoon groups were less preferred because of an overlap with working time). Both interventions were delivered by the same clinical psychologist (first author of the present study), officially trained in MBPI by Dr Alice Domar at Boston IVF, Waltham, Massachusetts, and experienced in psychological group leadership.

The interventions were designed in a way that the presumed therapeutic effect of certain MBPI elements could be tested. Therefore, the treatment arms contained some elements that were the same for both groups and other elements that were only used in one or the other group (Table 1). The common constituents served to make the control intervention, too, worthwhile and beneficial for a person affected by infertility, by focusing on topics such as psychoeducation on MAR procedures, the relationship between stress and infertility, and a fertility-promoting lifestyle (Domar *et al.*, 1990; Barbieri *et al.*, 2000). The distinctive elements of the MBPI fell into three categories: (1) CB therapy techniques, (2) stress management exercises, and (3) positive psychology. Cognitive techniques included the identification and restructuring of unhelpful infertility-related thoughts such as *I will never have a baby* or *I am damaged goods* or *Nothing works for me*, and the recognition of cognitive distortions behind these thoughts, such as catastrophizing, labelling, or overgeneralization. These methods have been proved to help patients with various psychiatric conditions understand the relationship between their emotions and irrational ideas, and train themselves in questioning these thoughts, which eventually results in less painful feelings (Clark, 2013). Stress management training consisted of an assertiveness exercise and formal, structured types of relaxation, such as autogenic training, progressive relaxation, and mindfulness meditation, since the regular performance of these practices has been shown to reduce anxiety (Manzoni *et al.*, 2008). The positive psychology task included was the use of gratitude diaries, shown elsewhere to be associated with well-being (Portocarrero *et al.*, 2020). A last distinguishing element of the MBPI was the assignment of home tasks, along with practical advice on how to incorporate relaxation exercises into everyday life.

**Table 1.** Comparative overview of the two interventions.

		Content	
Session	Content type	MBPI	FS
**1** ^a^	**Education**	MAR procedures and their physiological and psychological effects	

	**Activity**	introduction of group members; goals and mechanics of programme	

	**Relaxation**	diaphragmatic breathing	

	**Homework**	home practice of diaphragmatic breathing	–

**2**	**Education**	the physiology of stress and the relaxation response, the relationship between stress and the reproductive system	

	**Activity**	gratitude exercise	free interactional discussion on how group members relax

	**Relaxation**	mini-relaxation exercises	rectangle breathing technique

	**Homework**	home practice of gratitude and mini-relaxation exercises	–

**3**	**Education**	impact of lifestyle (nutrition, exercise) on infertility; evidence-based CAM methods	

	**Activity**	healthy diet quiz; sample diary on diet, exercise, and relaxation	free interactional discussion on what group members do to stay healthy

	**Relaxation**	raising mindfulness exercise	watching nature video

	**Homework**	starting progress diary; doing mindful activities	–

**4**	**Education**	–	typical emotions in infertility; the impact of infertility on self-esteem

	**Activity**	24-h time pie compared to personal list of pleasant activities	free interactional discussion on personal emotional reactions

	**Relaxation**	progressive relaxation	listening to relaxing music

	**Homework**	home practice of progressive relaxation	–

**5**	**Education**	self-empathy, nurturance, and compassion	

	**Activity**	women’s fertility yoga (with invited yoga instructor)	

	**Relaxation**	body scan	short resting time

	**Homework**	daily performance of nice activities	–

**6**	**Education**	the cognitive model on the relationship between thoughts, emotions, and behaviour	the effect of infertility on family relations and friendships

	**Activity**	cognitive restructuring (five-to-seven-column thought records)	free discussion on infertility-specific experiences with family members and friends

	**Relaxation**	autogenic training	head and face self-massage

	**Homework**	keeping thought records, home practice of cognitive restructuring	–

**7** ^a^	**Activity**	active listening exercise with partners	

	**Activity**	couples yoga (with invited yoga instructor)	

	**Relaxation**	meditation exercise	short resting time

	**Homework**	stocktaking exercise	–

**8**	**Education**	cognitive distortions	infertility and the workplace, reconciling work, and fertility treatments

	**Activity**	recognition of cognitive distortions; cognitive restructuring continued	free discussion on infertility-specific experiences with superiors and colleagues

	**Invited speakers**	2 prior group participants having chosen gamete donation vs adoption—questions and answers	

	**Relaxation**	guided imagery exercise	–

	**Homework**	home practice of cognitive distortion recognition	–

**9** ^a^	**Education**	the effect of infertility on partner relationships	

	**Activity (men)**	mini-support group with male therapist	

	**Activity (women)**	expressive writing exercise	free discussion on infertility-related partnership difficulties
	**Homework**	continue expressive writing for 3 more days	–

**10**	**Closure**	summary and review, follow-up information	

	**Activity**	revision of initial personal programme goals, saying goodbye	

	**Activity**	assertiveness exercise; goal setting exercise	free discussion on infertility and spirituality

	**Relaxation**	guided imagery exercise	–

All sessions lasted for 2 h and 15 min in both arms. Shared content elements in the two conditions are presented in merged cells. MBPI, Mind/Body Programme for Infertility; FS, Fertility Support group; MAR, medically assisted reproduction; CAM, complementary and alternative medicine.

a Partners present at session.

The design of the FS group was conceived in a way that it resembled the MBPI group as much as possible, with the exception of the ingredients of interest listed above. Psychoeducation was offered, but not followed by targeted exercises, and home practice was not overtly encouraged. Instead, free interactions and discussions were facilitated on the topics touched upon. Except for two breathing exercises, relaxation in the FS group was ‘informal’, such as head and face self-massage, listening to relaxing music, watching nature videos, and talking about self-nurture. The leadership style tended to be permissive, as opposed to the structured and directive methods applied in the MBPI group.

Participants of both groups filled the same psychological test battery within two to three weeks of programme completion (T2). Women were then followed for their MAR interventions and pregnancies until the end of the trial (T3), with the first groups having a two-and-a-half-year, and the last groups a half-year, follow-up. Yet, since MBPI and FS groups ran in a parallel manner, the mean follow-up time was the same in the two conditions. MAR interventions during follow-up included IUI and IVF, with or without ICSI. Cancelled cycles and frozen embryo transfers (FETs) were also counted in the ‘IVF’ category. Those discontinuing treatment were also followed if they attended at least half of the sessions. The T3 questionnaires did not contain psychological data.

### Primary outcome

Fertility-related QoL was assessed with the Fertility Quality of Life Scale (FertiQoL) (Boivin *et al.*, 2011b; Szigeti *et al.*, 2022). The 36-item instrument contains two general items, one on overall physical health and one on QoL satisfaction. The remaining 34 items are divided into a core section related to personal and interpersonal QoL (Core FertiQol), with items such as ‘Do you feel able to cope with your fertility problems?’ or ‘Do you feel uncomfortable attending social situations like holidays and celebrations because of your fertility problems?’, and an optional section related to treatment QoL (Treatment FertiQol), with items such as ‘How would you rate the surgery and/or medical treatment(s) you have received’. Core FertiQoL is made up of four subscales: Emotional, Mind-body, Relational, and Social subscales. Treatment FertiQoL comprises two subscales: Environment and Treatment tolerability. Response formats follow 5-point Likert scales. All scale scores range between 0 and 100, with higher scores indicative of better fertility-specific QoL. Internal reliability was 0.84 for the Core and 0.64 for the Treatment module scale, and ranged between 0.69 and 0.81 for the Core and 0.58 and 0.69 for the Treatment subscales.

As a diversion from the registered protocol, results on the SCREENIVF questionnaire (Verhaak *et al.*, 2010) as outcomes were not included in the study because of a delay in the Hungarian validation process.

### Secondary outcomes

Clinical pregnancy data were gathered as reported by patients on request. As an improvement in relation to the registered trial protocol, suggested specifically for reporting on infertility treatments, live births were also followed up (The Harbin Consensus Conference Workshop Group *et al.*, 2014).

Infertility-specific stress was measured with the Copenhagen Multi-centre Psychosocial Infertility (COMPI) Research Programme scales (Schmidt *et al.*, 2003, 2005; Pápay *et al.*, 2013), developed by the Copenhagen Multi-centre Psychosocial Infertility Research Programme launched in Denmark in 2000 (Schmidt, 2006). The COMPI Fertility Problem Stress Scales (COMPI FPSS; 14 items) measure the amount of stress caused by the fertility problem on three domains: personal (e.g. ‘My infertility problem has ruined my life’), marital (e.g. ‘My spouse/partner relationship is in crisis’), and social (e.g. ‘How stressful is it for you to meet people who have children?’), with responses given on 4- and 5-point Likert-scales. Results can range from 14 to 60 points, where higher scores indicate more stress. In the present sample, Cronbach-alpha value was 0.82 for the total Fertility Problem Stress Scale, and ranged between 0.61 and 0.79 for the FPSS subscales.

Depression was measured with the Beck Depression Inventory (BDI) (Beck *et al.*, 1961; Kopp *et al.*, 1990), which contains 21 items with responses on 4-step Likert scales such as from ‘I am not sad’ to ‘I am so sad and unhappy that I can’t stand it’, assessing symptoms of depression such as pessimism, lack of satisfaction, guilt, social withdrawal, being indecisive, inhibition from work, sleep disturbances, fatigue, and somatic preoccupation. Results can range from 0 to 63. Conventional cut-off scores on the BDI result in the following categories: normal range (0–9 points), mild (10–19 points), moderate (20–29 points), and severe depression (30–63 points). The nonclinical/clinical cut-off of 18/19 is routinely applied in Hungarian studies (Kopp and Skrabski, 1992). In the present sample, the questionnaire yielded a Cronbach alpha score of 0.87.

Anxiety was recorded with the Spielberger State-Trait Anxiety Inventory (STAI) (Spielberger *et al.*, 1970; Sipos and Sipos, 1983), which assesses anxiety on two 20-item scales: the State scale (STAI-S), measuring transient states of subjective fear, tension, and vegetative excitement, with items such as ‘I am tense’ and ‘I feel calm’, and the Trait scale (STAI-T), including statements such as ‘I worry too much over something that really doesn’t matter’ and ‘I am a steady person’, capturing a more stable tendency of an individual to get anxious. Both parts of the questionnaire are answered on 4-point Likert scales. Results on both scales can range from 20 to 80, where a higher score indicates greater levels of anxiety. In this study, only the Trait scale was utilized, based on findings rendering it as measuring negative affectivity in general (Knowles and Olatunji, 2020) or vulnerability to psychological disorders (Balsamo *et al.*, 2013), rather than an immutable personality trait (Zinbarg *et al.*, 2008). The STAI-T has been successfully used for capturing psychological changes following interventions in infertility (e.g. Chan *et al.*, 2012). In our sample, the scale had a Cronbach alpha value of 0.89.

General stress levels were gauged with the Short Stress Scale (SSS) (Purebl *et al.*, 2006) conceived to identify elements of cognitive, emotional, and behavioural stress reactions, rather than emotional states related to stress, such as anxiety or depression. The 26-item questionnaire mostly originates from the Hungarian version (Rózsa *et al.*, 2005) of the Brief Stress and Coping Inventory (Rahe *et al.*, 2000; e.g. ‘I am often pressed for time’), with five extra items based on semi-structured interviews with stress management training participants (e.g. ‘I have trouble sleeping’). Items are given yes-or-no (0- or 1-point) answers and, when added up, they yield a score between 0 and 26, where higher scores indicate more stress. In our sample, the scale had a Cronbach alpha value of 0.75.

### Statistical methods

Statistical analyses were performed with IBM SPSS for Windows, v20.0 (IBM Corp, 2011) and the lme4 package developed for R (Bates *et al.*, 2015). For continuous variables, after checking on the normality of distribution with Shapiro–Wilk tests, Welch’s *t*-tests or Wilcoxon signed rank tests were carried out to look for baseline test differences between the two groups. For categorical variables, Pearson chi-square (χ^2^) tests were used for comparison purposes.

The effect of the treatment on psychological parameters, measured both at baseline and after the intervention, was estimated using linear mixed effect models, which have been found to be more useful than mixed-model ANOVAs in repeated measures studies where multiple observations per experimental unit may not be independent of each other, and where unexplained error may not be the only source of random variability (Oberg and Mahoney, 2007). Group type (intervention or control), measurement occasion (baseline or post-intervention), and their interactions were introduced in the models as predictors, and a random intercept was used per participant. Time main effect refers to an aggregated pre-post change in psychological scores, irrespective of group membership. Group main effect refers to differences between group mean scores in bulk, regardless of measurement occasion. Group-by-Time interaction effect refers to group differences in the magnitude of pre-post score change, this being the key variable in the study. Cohen’s *d* effect sizes were calculated both for changes in mean scores from baseline for each group separately (time effect), and for differences in differences, i.e. between-group contrasts of within-person changes in relevant scores (group-by-time effect). Effect sizes were considered small if falling between 0.20 and 0.49, medium if between 0.50 and 0.79, and large if above 0.80.

The effect of the treatment on pregnancy-related outcomes, by design only available after the treatment, was estimated in logistic regression models. A statistically significant treatment group difference in the odds of successful fertility-related milestones was interpreted as evidence for the efficacy of treatment over control. All analyses were by original assigned groups.

Sociodemographic quasi-independent variables such as age, residence, financial situation, etc were checked for between-group differences so that they could be introduced as covariates in the model if any disparity occurred. For all models, *P*-values were estimated using Satterthwaite’s approximation implemented in the lmerTest R package.

## Results

**Figure 1.**
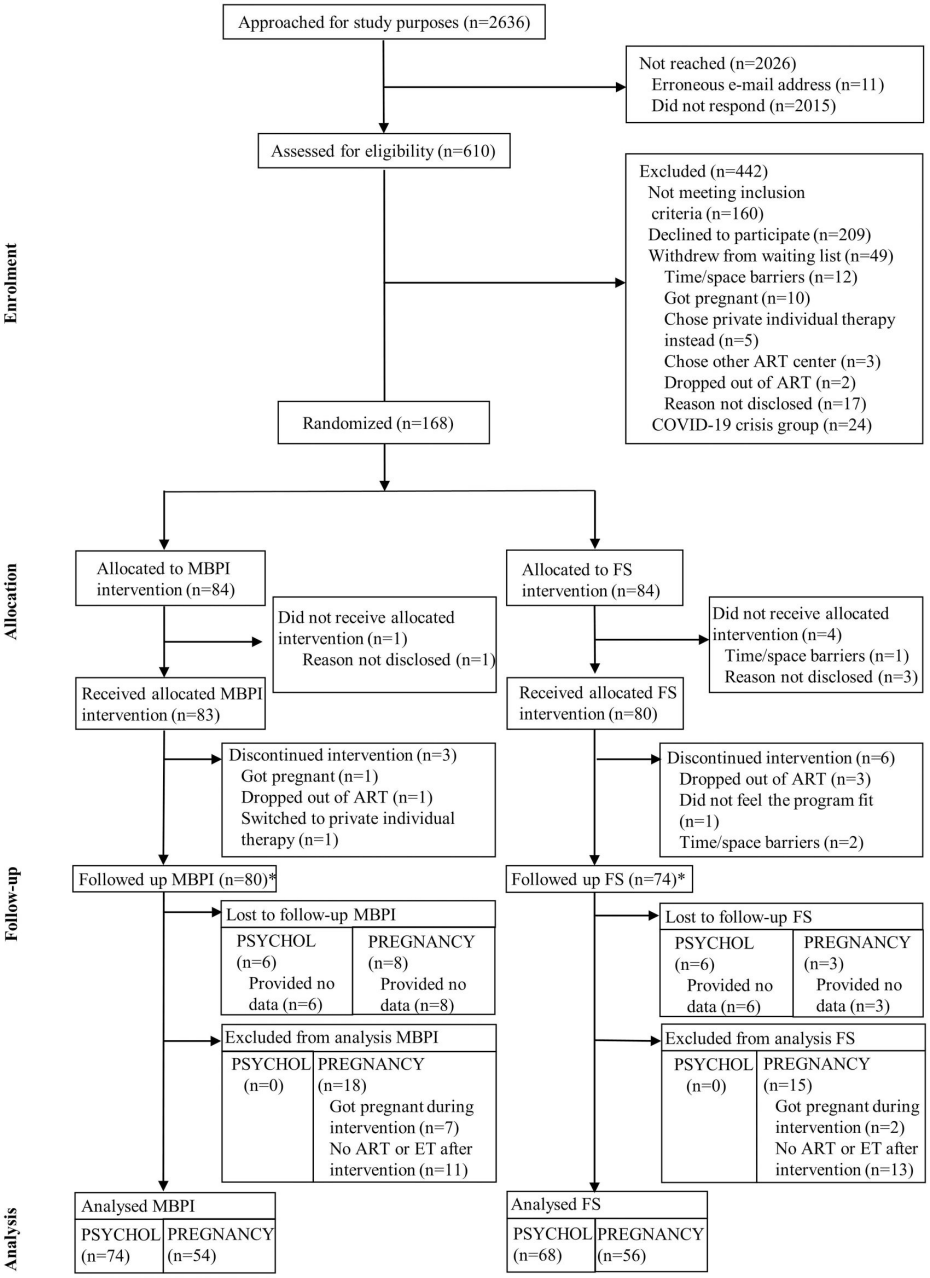
**CONSORT flowchart of participant recruitment and assignment**. ART, assisted reproductive treatment; ET, embryo transfer; FS, Fertility Support group; MBPI, Mind/Body Programme for Infertility group. *Followed up for either psychological or pregnancy outcomes, or both.

Out of the 2636 patients approached, 610 were assessed, 168 were randomized, and 154 (80 MBPI and 74 FS) were included in the analysis (Fig. 1). There were 74 MBPI and 68 FS group members were analysed for psychological outcomes, and 54 MS and 56 FS patients analysed for pregnancy outcomes.

**Figure 2.**
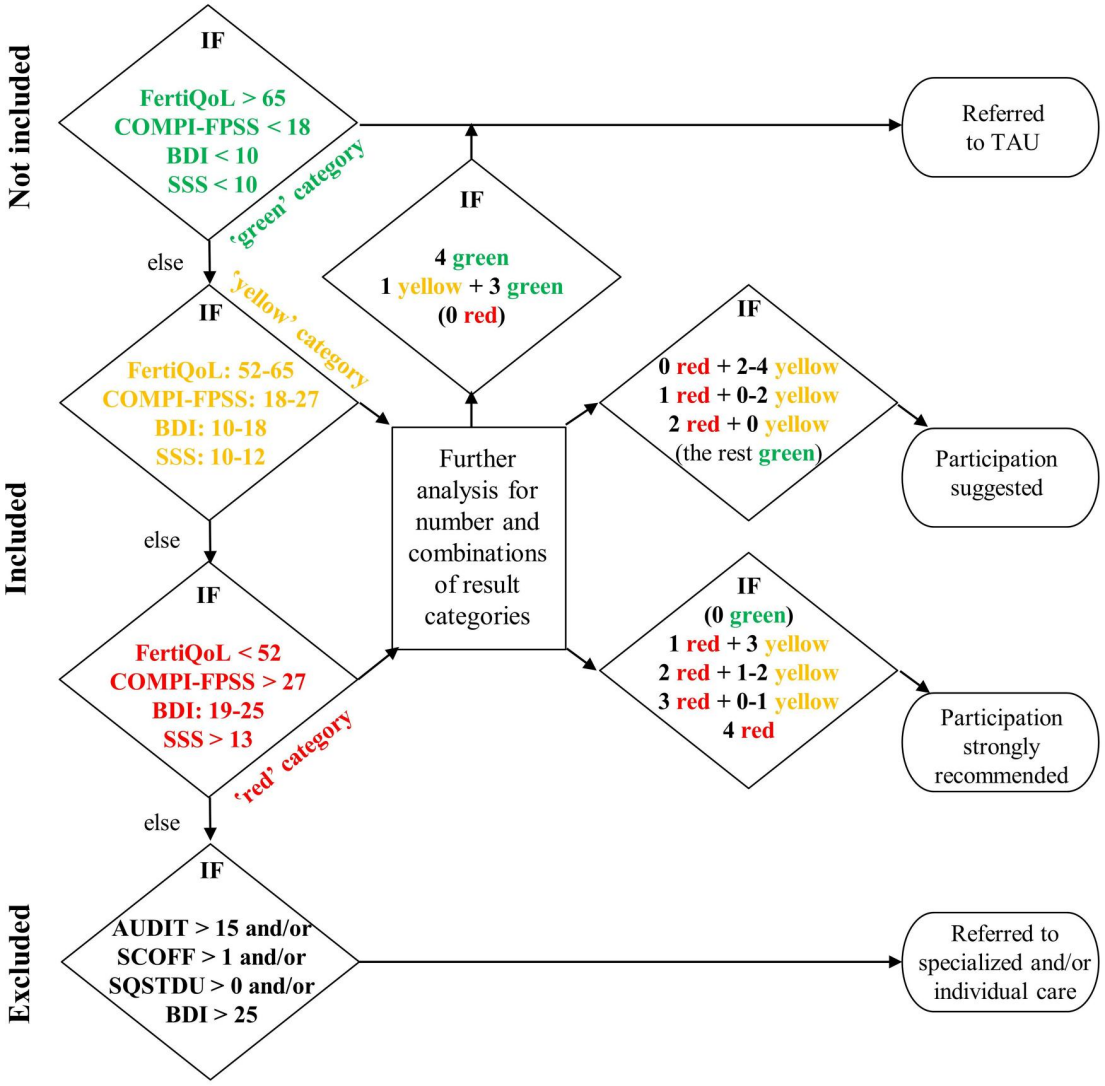
**Decision diagram for psychological eligibility**. AUDIT, Alcohol Use Disorders Identification Test; BDI, Beck Depression Inventory; COMPI-FPSS, COMPI Fertility Problem Stress Scales; FertiQoL, Fertility Quality of Life tool; SCOFF, screening tool for eating disorders; SQSTDU, Single-Question Screening Test for Drug Use; SSS, Short Stress Scale; TAU, treatment as usual.

A complex eligibility procedure was developed based on a combination of results on a number of psychological tools. Figure 2 lays out the selection procedure along the following principles: BDI cut-off values follow largely accepted international severity ranges, as suggested by Beck *et al.* (1996); Core FertiQoL thresholds are identical with those proposed in its Hungarian validation study, showing systematic covariance with BDI ranges (Szigeti *et al.*, 2022) and closely corresponding to cut-points suggested in other studies (Aarts *et al.*, 2011; Dural *et al.*, 2016); COMPI-FPSS cut-offs are based on mean ± 1 standard deviation values in its first-use Hungarian study (Pápay *et al.*, 2013); and SSS cut-offs were proposed by the developers of the scale (Purebl *et al.*, 2006). The procedure resulted in four categories of patients: (1) those in a relatively good mental state, referred to treatment as usual (‘green category’); (2) those mildly affected, offered to participate (‘yellow category’); (3) those moderately affected, strongly asked to participate (‘red category’); and (4) those suffering from severe depression, eating or substance disorder, referred to specialized care.

Out of the 450 patients who met the inclusion criteria and were offered to join the psychological programme after screening, 46.4% (209) were not interested in participating, 10.9% (49) withdrew from the waiting list for mostly known reasons, 5.3% (24) were excluded due to COVID-19, while the remaining 37.3% (168) were randomized. The majority of the randomized patients (98.8% (83) of those allocated in the MBPI group (84) and 95.2% (80) of those assigned to the FS group (84)) received the intervention. Put in another way, out of the patients approached for the purposes of the study (2636), 17% (450) were found to be psychologically vulnerable at a mild-to-moderate level, and 6.2% (163) attended the psychological programme.

Eight MBPI and eight FS groups with 8–12 women per group ran in a parallel manner between June 2020 and December 2022. Out of the 10 sessions, participants attended a mean (M) of 8.88 (SD = 1.38). The attrition rate, defined as leaving the group and not returning, was 5.5% (9/163) on average: 3.6% (3/83) in the MBPI group and 7.5% in the FS group (6/80). Based on an intention-to-treat analysis, loss to follow-up (LTFU) was 7.1% (6) for psychological and 9.5% (8) for pregnancy data in the MBPI group (84), and 7.1% (6) for psychological and 3.6% (3) for pregnancy data in the FS group (84), as shown in Fig. 1.

As for their sociodemographic and other baseline characteristics (Table 2), participants were in their mid-30s, with those assigned to the MBPI group turning out to be significantly younger than those in the FS group (*Z* = −3.286, *P* < 0.001). The majority lived in secure relationships, cohabiting or married. Most of them held higher education degrees, lived in urban areas, were employed, and perceived their financial situation as above average. Almost all had primary infertility which had lasted for 3.5 years on average. Aetiology was varied, with one-third being of female origin, another third unexplained or (yet) unknown, the rest being of male or combined origin. Patients were predominantly in the *in vitro* treatment phase. No pre-intervention differences were detected in five of the psychological variables measured (Table 3), except the Treatment FertiQoL score was significantly lower in the FS than in the MBPI group (*t* = −2.180; *P*=0.031; Table 3). That is, apart from age and treatment-related QoL, all baseline data were similar in the two groups, so the randomization process was predominantly successful.

**Table 2.** Descriptive statistics for sociodemographic and medical characteristics at baseline for MBPI (n* *=* *80) and FS (n* *=* *74) groups.

Characteristic	MBPI	FS	Test statistic
Sociodemographic variables			
Age, M (SD), years	34.34 (4.75)	36.89 (3.96)	***t*=−3.286**
Marital status			χ^2^ = 3.441
Married, N (%)	65 (81.3)	57 (77.1)	
Cohabiting, N (%)	15 (18.8)	15 (20.3)	
Single, N (%)	0 (0.0)	2 (2.7)	
Number of existing children			χ^2^ = 3.247
None, N (%)	79 (98.8)	69 (93.2)	
One, N (%)	1 (1.3)	4 (5.4)	
Two, N (%)	0 (0.0)	1 (1.4)	
Residence			χ^2^ = 5.581
Capital, N (%)	52 (65.0)	44 (59.5)	
City, N (%)	2 (2.5)	7 (9.5)	
Town, N (%)	18 (22.5)	17 (24.4)	
Village, N (%)	7 (8.8)	5 (6.8)	
Other, N (%)	1 (1.3)	0 (0.0)	
Education			χ^2^ = 1.620
Primary, N (%)	0 (0.0)	0 (0.0)	
Secondary vocational, N (%)	2 (2.5)	1 (1.4)	
Secondary GCE, N (%)	5 (6.3)	8 (10.8)	
Postgraduate vocational, N (%)	8 (10.0)	6 (8.1)	
College/university, N (%)	59 (73.8)	55 (74.3)	
Doctoral studies, N (%)	6 (7.5)	4 (5.4)	
Employment status/Position			χ^2^ = 5.742
Employed, subordinate, N (%)	54 (67.5)	55 (74.4)	
Employed, mid-level, N (%)	10 (12.5)	10 (13.5)	
Executive/Manager, N (%)	3 (3.8)	4 (5.4)	
Self-employed, N (%)	11 (13.8)	4 (5.4)	
Unemployed, N (%)	1 (1.3)	0 (0.0)	
Other, N (%)	1 (1.3)	1 (1.4)	
Health information			
Body Mass Index, M (SD)	23.33 (5.15)	24.17 (4.84)	*t*=−1.539
Underweight, < 18.50, N (%)	5 (6.3)	2 (2.7)	
Healthy weight, 18.50–24.99, N (%)	53 (66.3)	45 (60.8)	
Overweight, 25.00–29.90, N (%)	12 (15.0)	19 (25.7)	
Obese, >29.90, N (%)	8 (10.0)	8 (10.8)	
Missing, N (%)	2 (2.6)	0 (0.0)	
Duration of infertility, M (SD), months	37.54 (25.38)	39.86 (26.75)	*t*=−0.830
Aetiology of infertility			χ^2^ = 6.192
Female, N (%)	26 (32.5)	24 (32.4)	
Male, N (%)	10 (12.5)	3 (4.1)	
Combined, N (%)	17 (21.3)	25 (33.8)	
Unexplained, N (%)	18 (22.5)	18 (24.3)	
Does not know/No work-up yet, N (%)	9 (11.3)	4 (5.4)	
Infertility treatment stage			χ^2^ = 1.181
Work-up or *in vivo*, N (%)	12 (15.0)	16 (21.6)	
IUI, N (%)	13 (16.3)	12 (16.2)	
IVF, N (%)	55 (68.8)	46 (62.2)	

Welch’s *t*-tests for continuous data, chi-square for categorical data. None of the tests were statistically significant, except for age (in bold; *P*<0.001). MBPI, Mind/Body Programme for Infertility group; FS, Fertility Support group; M, mean; SD, standard deviation; N, number; %, percentage; IUI, intrauterine insemination; IVF, *in vitro* fertilization.

**Table 3.** Descriptive statistics for differences in baseline psychological scores in MBPI (n* *=* *80) and FS (n* *=* *74) groups.

Psychological test	Test statistic	*P*-value	Mean difference	SE difference
Core FertiQoL	−0.191	0.849	−0.377	1.974
Treatment FertiQoL	2.180	0.031*	4.14	1.903
COMPI-FPSS	0.579	0.564	0.651	1.125
BDI	−0.385	0.701	−0.476	1.238
STAI-T	−0.633	0.527	−1.034	1.633
SSS	0.024	0.981	0.018	0.743

Welch’s independent samples *t*-tests for each variable. None of the tests were statistically significant, except for treatment-related quality of life (in bold). MBPI, Mind/Body Programme for Infertility group; FS, Fertility Support group; SE, standard error; Core FertiQoL, Core module of the Fertility Quality of Life tool; Treatment FertiQoL, Treatment module of the Fertility Quality of Life tool; COMPI-FPSS, COMPI Fertility Problem Stress Scale; BDI, Beck Depression Inventory; STAI-T, Trait subscale of the Spielberger State-Trait Anxiety Inventory; SSS, Short Stress Scale.* Significant at the 0.05 level.

Out of the patients followed up for obstetric outcomes between T2 and T3, 75.0% (54) of the MBPI group (72) and 78.8% (56) of the FS group members (71) received MAR treatments (Table 4). While there was no statistically significant difference between the overall number of MAR interventions received, significantly fewer MBPI than FS patients received IVF (instead of IUI), probably because they were younger. No significant differences were detected between the two groups in any kind of medical outcome, including the number of blastocyst-stage embryos, MAR pregnancies, spontaneous pregnancies, and live births. The overall average cumulative MAR pregnancy rate after an average of 1.72 cycles was 50.90% (calculated for only patients under MAR treatment). The overall average cumulative rates of any pregnancy and any live birth were 50.35% and 34.27%, respectively, among patients followed up, and 42.86% and 29.17%, respectively, based on an intent-to-treat analysis, with the conservative interpretation that none of the patients who did not receive the allocated psychological treatment or discontinued it, or were lost to follow-up, became pregnant (Table 4).

**Table 4.** MAR-related data and between-group comparisons of obstetric outcomes at follow-up.

	Patients followed up for obstetric outcomes				
MAR-related data	**MBPI** (N* *=* *72)	**FS** (N* *=* *71)	χ^2^	*P*-value	**MBPI+FS** (N* *=* *143)
Patients receiving MAR, N	54	56	1.259	0.262	110
MAR cycles, N	92	101	0.627	0.428	193
IUI cycles, N	12	6	0.603	0.895	18
IVF cycles,^a^^,b^ N	80	95	10.379	**0.034** *	175
MAR cycles/patient, M (range)	1.69 (1–5)	1.76 (1–4)			1.72 (1–5)
Number of Day 5 embryos at first IVF, M (SD)	3.91 (3.89)	3.38 (2.41)	11.295	0.504	3.64 (3.20)
Number of Day 5 embryos at second IVF, M (SD)	3.18 (2.55)	2.56 (2.15)	1.417	0.994	2.86 (2.34)
MAR pregnancies, N	29	27	0.716	0.397	56
IUI pregnancies, N	3	2	0.008	0.928	5
IVF pregnancies, N	26	25	0.697	0.404	51
Spontaneous pregnancies, N	9	7	0.251	0.616	16
All pregnancies, N	38	34			72
IUI pregnancy rate/started cycle^b^	25.00%	33.33%			27.77%
IVF pregnancy rate/started cycle^b^	32.50%	26.31%			29.14%
Cumulative MAR pregnancy rate^c^	53.70%	48.21%			50.90%
Cumulative all pregnancy rate (all patients folllowed up)	52.78%	47.89%			50.35%
Cumulative all pregnancy rate (ITT analysis)^d^	45.24%	40.48%			42.86%
Live births, N	23	26	0.076	0.783	49
Cumulative MAR live birth rate	39.58%	41.82%			40.77%
Cumulative live birth rates (all patients followed up)	31.94%	36.62%			34.27%
Cumulative live birth rate (ITT analysis)^d^	27.38%	30.95%			29.17%

MAR, medically assisted reproduction; MBPI, Mind/Body Programme for Infertility group; FS, Fertility Support group; N, Number; M, Mean; SD, Standard deviation; %, percentage; IUI, intrauterine insemination; IVF, *in vitro* fertilization; ITT, intention to treat.

a Frozen embryo transfer cycles included.

b Cycles not resulting in oocyte retrieval included.

c Only patients receiving MAR included; calculated for a 30-month follow-up.

d All patients and types of pregnancies included; calculated for a 30-month follow-up.

* Significant at the 0.05 level.

**Figure 3.**
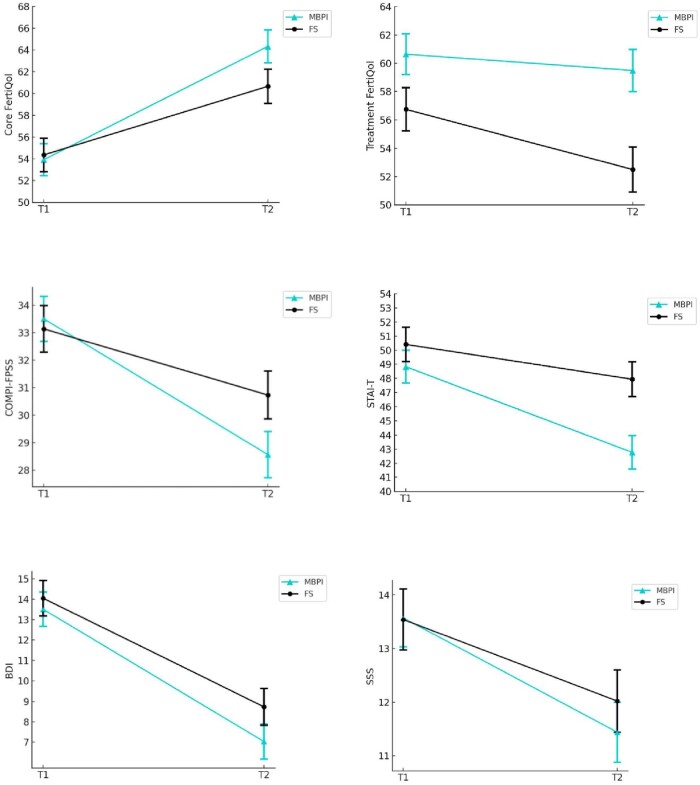
**Mean psychological score changes for the experimental (MBPI) and the control group (FS) across the two assessment times**. Error bars represent standard error of the mean. MBPI, Mind/Body Programme for Infertility group; FS, Fertility Support group. Core FertiQoL, Core module of the Fertility Quality of Life tool; Treatment FertiQoL, Treatment module of the Fertility Quality of Life tool; COMPI-FPSS, COMPI Fertility Problem Stress Scale; BDI, Beck Depression Inventory; STAI-T, Trait subscale of the Spielberger State-Trait Anxiety Inventory; SSS, Short Stress Scale.

To neutralize the age difference between the two groups, also because age is a well-known prognostic variable in the case of MAR success (Wang *et al.*, 2008), we adjusted for age in all analyses (Roberts and Torgerson, 1999). The linear mixed effect analysis revealed a significant Time (T1–T2) main effect in the case of all psychological outcomes, except for treatment-related QoL, with effect sizes predominantly medium to large in the MBPI group, and small to medium in the FS group, with the largest improvements in depression and fertility QoL in both groups (Table 5). No significant differences in Group main effects (MBPI vs FS, regardless of measurement time) were detected for the majority of the psychological variables. In the initial analysis, significant Group-by-Time interactions of small effect sizes were found in fertility-related QoL, infertility-specific stress, and trait anxiety, such that the pre-post changes in the MBPI group were more accentuated than those in the FS group (Fig. 3). Namely, there was a 4.11-point greater improvement in fertility-related QoL, a 2.54-point more powerful decrease in infertility-specific distress and a 3.60-point stronger reduction in trait anxiety in the MBPI than in the FS group (see Group×Time estimates, that is, differences in differences, in Table 5). After controlling family-wise error rate with the Holm–Bonferroni correction, however, the group-by-time interaction effect remained significant only in the case of trait anxiety. No interaction effect appeared in the case of either treatment-related QoL, depression, or general distress. Age had no independent effect on any psychological outcome.

**Table 5.** Linear mixed effects model statistics for psychological response variables in the MBPI and FS groups, adjusted for age.

			Core Ferti-QoL	Treat-ment Ferti-QoL	COMPI-FPSS	BDI	STAI-T	SSS
**Intercepts**	**MBPI**	**T1**	53.93	60.64	33.51	13.51	48.83	13.57
		**SE**	1.47	1.43	0.82	0.84	1.16	0.54
		**95% CI**	[51.02, 56.84]	[57.82, 63.47]	[31.90, 35.11]	[11.86, 15.16]	[46.55, 51.12]	[12.50, 14.64]
	**FS**	**T1**	54.37	56.75	33.14	14.05	50.41	13.54
		**SE**	1.54	1.52	0.85	0.87	1.21	0.57
		**95% CI**	[51.34, 57.40]	[53.75, 59.74]	[31.46, 34.81]	[12.32, 15.77]	[48.03, 52.80]	[12.42, 14.66]
**Slopes**	**MBPI**	**MD**	10.40	−1.16	−4.95	−6.48	−6.06	−2.13
		**95% CI**	[7.35, 13.02]	[−4.26, 2.46]	[−6.52, −3.37]	[−8.07, −4.77]	[−7.88, −4.29]	[−2.99, −1.20]
	**FS**	**MD**	6.29	−4.25	−2.41	−5.32	−2.47	−1.52
		**95% CI**	[3.50, 8.45]	[−8.60, −1.65]	[−3.89, −0.84]	[−7.00, −3.21]	[−4.25, −0.43]	[−2.45, −0.49]
**Fixed effects**	**Time**	**Estimate**	8.35	−2.70	−3.68	−5.90	−4.27	−1.83
		***P*-value**	<0.001***	0.024*	<0.001***	<0.001***	<0.001***	<0.001***
		**MBPI effect size**	0.69	−0.10	−0.64	−0.90	−0.56	−0.44
		**FS effect size**	0.54	−0.30	−0.34	−0.68	−0.24	−0.31
	**Group**	**Estimate**	−1.62	−5.44	0.90	1.12	3.38	0.28
		***P*-value**	0.416	0.003**	0.408	0.304	0.035*	0.708
	**Group×Time**	**Estimate (DD)**	4.11	−3.08	−2.54	−1.16	−3.60	−0.62
		**95% CI**	[0.42, 7.80]	[−7.72, 1.55]	[−4.68, 0.41]	[−3.61, 1.29]	[−6.16, −1.04]	[−1.91, 0.68]
		***P*-value**	**0.031** *	0.195	**0.021** *	0.355	**0.007** **	0.354
		**Adjusted**	0.124	0.582	0.105	0.708	**0.042** *	0.708
		***P*-value** ^a^						
		**DD effect size**	0.32	−0.20	−0.36	−0.13	−0.32	−0.13

Intercepts express baseline mean scores on the scales applied. Slopes express pre-post differences in mean scores. Group-by-time interaction parameter estimates express differences in mean group score changes, with the control group (FS) as a reference. Age was introduced as a confounding factor, and centred (values not shown). ‘Time’ refers to a pre-post main effect alone, irrespective of group membership. ‘Group’ refers to a group membership main effect alone, irrespective of measurement time. ‘Group×Time’ refers to the interactional effect of pre-post measurement and group membership, the result of interest in the study. Statistically significant effects of interest are set in bold.

MBPI, Mind/Body Programme for Infertility; FS, Fertility Support; CI, confidence interval; T1, Time 1; SE, standard error; MD, mean pre-post difference; DD, difference in differences; Core FertiQoL, Core module of the Fertility Quality of Life tool; Treatment FertiQoL, Treatment module of the Fertility Quality of Life tool; COMPI-FPSS, COMPI Fertility Problem Stress Scale; BDI, Beck Depression Inventory; STAI-T, Trait subscale of the Spielberger State-Trait Anxiety Inventory; SSS, Short Stress Scale.

a Holm–Bonferroni.

* Significant at the 0.05 level.

** Significant at the 0.01 level.

*** Significant at the 0.001 level.

The logistic regression showed no Group effect on MAR pregnancies, spontaneous pregnancies, or live births (Table 6). Age, however, had a marked significant main effect in each of the MAR-related outcomes, except for spontaneous pregnancies.

**Table 6.** Binary logistic regression model statistics for MAR response variables, with the control group (FS) as a reference.

Pregnancy outcomes	Predictive variable	Estimate	Standard error	Test statistic	*P*-value
MAR pregnancy	Age	0.169	0.055	−3.058	0.002**
	Group	−0.037	0.433	−0.086	0.931
Spontaneous pregnancy	Age	−0.127	0.080	−1.585	0.113
	Group	0.370	0.635	0.583	0.560
Any pregnancy	Age	0.147	0.045	3.256	0.001**
	Group	−0.271	0.376	−0.722	0.470
Live birth	Age	0.172	0.047	3.625	<0.001***
	Group	−0.583	0.406	−1.436	0.151

Age was introduced as a confounding factor. ‘Group’ refers to Mind/Body Programme for Infertility (MBPI) or Fertility Support group (FS) membership effect. MAR, medically assisted reproduction.

** Significant at the 0.01 level.

*** Significant at the 0.001 level.

As for adversities occurring during the trial, some participants reported deterioration in psychological wellbeing, caused by listening to the often difficult stories of other group members ahead of them on the pathway of MAR treatments. This, however, typically happened at the initial phase of the interventions, and was always temporary, never resulting in an actual adverse outcome.

## Discussion

The aim of the present study was to test the clinical effectiveness of the MBPI in relation to a partly matched fertility support programme on a sample of women undergoing MAR with higher-than-average stress levels, and to isolate the active ingredients of the MBPI. The MBPI intervention did not result in significantly greater improvements in the primary outcome, that is, fertility-specific QoL, or in most of the secondary outcomes, i.e. infertility stress, depression, and general distress, but it did lead to a statistically significant between-group difference in changes to trait anxiety. There were also no differences in MAR-treatment-related QoL or pregnancy status. Thus, it seems that the assumed active ingredients of the MPBI were successful in more strongly ameliorating trait anxiety but not the other constructs tested.

It is informative that the factors only present in the MBPI condition, namely, CB and stress management techniques, seem to have brought about an augmented improvement in trait anxiety compared to the other condition. Additionally, home assignments entail a greater involvement on the part of the patients, e.g. keeping records of unhelpful thoughts and making a habit out of neutralizing them, which may have also contributed to the larger effect. This is in line with a literature review concluding that, in the case of group cognitive therapy, specific factors are more influential than non-specific ones (Oei and Shuttlewood, 1996). Given that most interventions in the infertility field are a mixture of different methods, the effects of which are difficult to differentiate (Kremer *et al.*, 2023), it is of value that our research may have helped to identify a successful intervention’s active ingredients affecting trait anxiety. We have also learned from this study that the same elements did not bring extra benefits in other psychological domains. Although it seemed straightforward to assume that CB and formal relaxation elements enhance beneficial effects on infertility-specific stress, depression, and general stress as well, these may not, or not in themselves, be the active ingredients of the MBPI in these respects.

The common elements of the two programmes must have largely contributed to the considerable within-subjects effects, since both psychosocial interventions robustly ameliorated the mental state of women in MAR treatment along most constructs. The fact that both groups were led by the same psychologist may have controlled the therapist variable, but possibly introduced another common element in the two conditions. Additionally, non-specific factors must have been at work here, too. Attention, empathy, acceptance, emotional containment, and group support, together with the placebo effect, that is, the expectation for improvement, could also have played a part in the effectiveness of both groups.

The only measure that stagnated or, in the FS group, worsened, was treatment-related QoL. This may be due to the fact that the patients were still in the midst of stressful and often unpredictable MAR treatments, especially members of the FS group, who received more invasive treatment (more undergoing IVF instead of IUI).

Concerning pregnancy data, the lack of a TAU control group makes it impossible to find out whether the psychosocial interventions contributed to pregnancy chances or not. In our complex model, the effect of age was much stronger than that of the intervention, irrespective of its character, suggesting that psychological gains cannot overrule the relentlessness of biology. Nevertheless, both per-cycle and cumulative MAR pregnancy rates in our sample were higher than those reported in international data for the respective age groups and cycle numbers (Gnoth *et al.*, 2011; Tigges *et al.*, 2016), especially since cancelled cycles were also included in our calculations.

Our study has partly replicated the results of Domar et al.’ (2000a,b) RCTs in that the MBPI, then called a CB-group, led to better psychological results in certain constructs, but not to more pregnancies than the support group. What is new compared to the original RCTs is that (1) due to modifications in the design, more precise information was gathered on catalysts of change; (2) the tools applied allowed a differentiation between general and condition-specific effects; and (3) sample selection based on psychological state severity allowed us to test the method on patients most in need, thus demonstrating the clinical applicability of the MBPI. This aspect, namely, whether psychosocial interventions are effective in women with elevated anxiety and depression levels, was found as necessary to investigate in a recent meta-analysis (Kremer *et al.*, 2023).

Meta-analytic results are not unanimous about whether certain types of interventions are more effective than others in helping people with infertility. A lot of them point out the superiority of CB and mind-body approaches over others (Katyal *et al.*, 2021; Zhou *et al.*, 2021; Koochaksaraei *et al.*, 2023), while some find no clear-cut efficacy differences (Frederiksen *et al.*, 2015; Dube *at al*., 2023). The same is true for therapy formats: one study found that group formats have more convincing results than individual or couple formats for combined psychological outcomes (Frederiksen *et al.*, 2015), but others found similar efficacies (de Liz and Strauss, 2005; Dube *et al.*, 2023). Our results are in line with the body of literature corroborating the beneficial effects of group psychosocial interventions in general (Warne *et al.*, 2023), and CB and mind-body type groups in particular (Gaitzsch *et al.*, 2020; Ha and Ban, 2021), on the wellbeing of women dealing with infertility. Our result is in particular agreement with a meta-analysis detecting a favourable effect of psychosocial interventions on anxiety levels (de Liz and Strauss, 2005), and another one finding mind-body approaches particularly suitable for ameliorating anxiety (Zhou *et al.*, 2021), but not with a recent method-critical meta-analysis which found significant changes in depression, but not anxiety (Kremer *et al.*, 2023), although, as we have already expressed, tools such as the STAI-T may cover both constructs.

Not much is known about the differential effect of psychosocial interventions on general versus infertility-specific distress, with not more than one meta-analysis examining this aspect, and finding evidence for improvement in generic, but not in specific constructs (Frederiksen *et al.*, 2015). Here, the use of a wide range of psychological outcomes allowed us to examine the extra general versus condition-specific effects of the same intervention, and to reach a similar conclusion. That is, while fertility-related wellbeing improved robustly overall, no additional impact of the on-site and home exercises could be detected, despite their content-specificity, e.g. cognitive restructuring performed on negative thoughts central to unintended childlessness.

A serious caveat was initially raised by the fact that, although randomization was successful in other recorded variables, chance bias appeared in connection with age, such that the experimental group was significantly younger than the control group. Nonetheless, the results presented here included age adjustment. Age did have a strong effect on pregnancy rates in our sample, in harmony with previous findings that it is an independent factor in ART success (Dabbagh Rezaeiyeh *et al.*, 2022), but it did not influence psychological outcomes. Indeed, while age seems to play a part in premature discontinuation of psychotherapies (Swift and Greenberg, 2012), there is no conclusive evidence for it interfering with therapy success. The differences in therapy outcomes, if any, occur between the old and the young (Boswell *et al.*, 2016), and not within the age range of our sample.

From a sociodemographic point of view, our participants fit international trends of ART help-seeking ages in European countries (Chandra *et al.*, 2014), where having the first children tends to be postponed (Passet-Wittig and Greil, 2021), possibly resulting in age-related infertility. The sample showed fairly high socioeconomic status, also typical of patients in ART (Datta *et al.*, 2016). Marital status reflects Hungarian legislation, namely, that ART is allowed only for married or officially cohabitating opposite-sex couples and single women who have two independent expert opinions stating that they cannot have children otherwise.

The unfortunate coincidence of the trial period with the COVID-19 pandemic posed considerable challenges, such as accounting for the much-altered needs of the participants. With the threat of any-time treatment cancellations due to the emergence of a new wave or patients’ virus infection, with partners not allowed to accompany women to examinations and treatments, COVID-19 has magnified the stress of infertility (Irani *et al.*, 2022), and affected trial methodology as well. The pandemic deterred many patients from enrolling because of health anxieties, thus slowing down participant uptake. During the study period, couple sessions had to be held online, and COVID-specific stress had to be addressed, potentially altering the results.

The ratios of patients found to be psychologically vulnerable, and those interested and actually attending the psychological programme are comparable with the take-up of psychosocial services in the ART setting in previous studies (Spoletini *et al.*, 2022). The average session attendance rate (88.8%) was impressively high, and the attrition rate (5.5%) was low, as compared to that of psychological interventions in general, where the dropout is estimated to be 17% in efficacy studies and 26% in effectiveness studies (Swift and Greenberg, 2012), and to attrition rates in group settings ranging between 25% and 31% (Hofmann and Suvak, 2006; Gulamani *et al.*, 2020). This points to the feasibility of the intervention, and to the remarkably high motivation of women facing infertility to raise in all possible ways their chances of having a child. Attendance by partners was much lower: although most of the patients lived in long-term relationships, only about half of the men were present at the meetings intended for couples, with typical reasons being long working hours and a lower interest in psychological services, consistent with the well-known reduced utilization of mental health care among men as compared to women (Mackenzie *et al.*, 2006; Terlizzi and Zablotsky, 2020). Although the attrition rate in the FS group was higher than in the MBPI group, the ratio was reversed in the follow up. The lost-to-follow-up rate was lower than 10% in all subgroups, introducing selection bias at an acceptable degree (Dettori, 2011).

As for adverse events, we observed the initial worsening of some patients confronted for the first time with the possible pitfalls of the MAR journey. However, these hard feelings disappeared very soon, often followed by a relativization of the patients’ own problems as compared to what they heard from others, and giving way to a sense of contentment about the useful information the shared stories contained. Therefore, the psychological benefits of the trial clearly outweighed its harms.

One of the strengths of our study lies in the heterogeneity of the sample in terms of age and the type, duration and treatment stage of infertility, as a fair reflection of the clinical population in the ART setting. Another strong point is the very low attrition and acceptable LTFU rate, all of which raise the external validity and generalizability of our trial. Additionally, the fairly long and extensive follow-up made the reporting of live birth rates possible, in accordance with IMPRINT, an addition to the CONSORT statement to improve the quality of reporting clinical trials of infertility treatments (The Harbin Consensus Conference Workshop Group *et al.*, 2014). Finally, the study is gap-filling in the Hungarian setting, where routine psychological care is practically missing in the MAR setting, and no data on the MBPI have been available.

The study has some shortcomings, too. First, since the groups started every 3–4 months, for some patients there was a time lapse between baseline measurement and programme initiation, during which their psychological status may have changed. Second, there was a difference in the rate of pregnancy follow-up in the two groups, which may have biased the results. Third, at the several-month follow-up time point only medical and no psychological data were registered, nor was there a measurement of whether the patients adhered to the exercises, relaxation or other, learned during the programme. Thus, the long-term psychological effects are not known in either treatment arm, nor is the durability of the added MBPI value. Fourth, biological factors other than age, aetiology and duration of infertility may have confounded study results. Fifth, the single-centred design of the study, the 23% initial response rate and the rather large number difference between patients invited and those included in the study may lower the generalizability of our RCT. Lastly, men were not included at all stages, with some of the attenders expressing their regrets over this. Future studies should further explore these topics, take even more possible confounders into consideration, apply multi-point measurements, and use larger samples and alternative analyses, e.g. Bayesian multilevel modelling. Also, men should be more extensively included in study samples, to balance the inequalities in the information available on the psychological adjustment to MAR of the two genders.

## Conclusion

The MBPI had similar results in terms of fertility-specific psychological outcomes and pregnancies, but performed better than a partly matched support group in terms of an important general psychological outcome, trait anxiety, in a female population living with infertility. Besides the overall benefit, CB, relaxation, and some other stress management techniques complemented with home tasks have been identified as the specific factors of the MBPI causing the change surplus in trait anxiety, but not in depression, general stress and fertility-related wellbeing. Further research design modifications would be necessary to identify other specific factors of the MBPI. Now that, besides the known efficacy and feasibility of the MBPI, its overall effectiveness has also been established, testing its efficiency is also timely, e.g. to check whether the same effect can be reached in an even more economical way, say, with six sessions instead of ten. All in all, our study showed that the MBPI works well not only in controlled, experimental conditions, but also in routine MAR practice, and is suitable for being introduced as a cost-effective, low-intensity psychological intervention, within the framework of stepped care.

## Data Availability

The data underlying this article cannot be shared publicly for the privacy of individuals that participated in the study, but will be shared on reasonable request to the corresponding author.
